# Water Pores in Planar Lipid Bilayers at Fast and Slow Rise of Transmembrane Voltage

**DOI:** 10.3390/membranes11040263

**Published:** 2021-04-05

**Authors:** Alenka Maček Lebar, Damijan Miklavčič, Malgorzata Kotulska, Peter Kramar

**Affiliations:** 1 Faculty of Electrical Engineering, University of Ljubljana, 1000 Ljubljana, Slovenia; damijan.miklavcic@fe.uni-lj.si; 2 Department of Biomedical Engineering, Faculty of Fundamental Problems of Technology, Wroclaw University of Science and Technology, 50-370 Wrocław, Poland; malgorzata.kotulska@pwr.edu.pl

**Keywords:** planar lipid bilayer, capacitance, voltage breakdown, water pores, hydrophobic pores

## Abstract

Basic understanding of the barrier properties of biological membranes can be obtained by studying model systems, such as planar lipid bilayers. Here, we study water pores in planar lipid bilayers in the presence of transmembrane voltage. Planar lipid bilayers were exposed to fast and slow linearly increasing voltage and current signals. We measured the capacitance, breakdown voltage, and rupture time of planar lipid bilayers composed of 1-pamitoyl 2-oleoyl phosphatidylcholine (POPC), 1-pamitoyl 2-oleoyl phosphatidylserine (POPS), and a mixture of both lipids in a 1:1 ratio. Based on the measurements, we evaluated the change in the capacitance of the planar lipid bilayer corresponding to water pores, the radius of water pores at membrane rupture, and the fraction of the area of the planar lipid bilayer occupied by water pores.planar lipid bilayer capacitance, which corresponds to water pores, water pore radius at the membrane rupture, and a fraction of the planar lipid bilayer area occupied by water pores. The estimated pore radii determining the rupture of the planar lipid bilayer upon fast build-up of transmembrane voltage are 0.101 nm, 0.110 nm, and 0.106 nm for membranes composed of POPC, POPS, and POPC:POPS, respectively. The fraction of the surface occupied by water pores at the moment of rupture of the planar lipid bilayer The fraction of an area that is occupied by water pores at the moment of planar lipid bilayer rupture is in the range of 0.1–1.8%.

## 1. Introduction

Biological membranes, the barriers that envelope the cell and its inner organelles, play an important role in the physiology and functionality of cells. Although biological membranes are composed of lipids, proteins, and small amounts of carbohydrates, the barrier function is assured by the thin lipid bilayer. Basic understanding of barrier properties of biological membranes can be obtained by investigating model systems, such as artificial liposomes or vesicles, which mimic the geometry and size of the cell membrane but are devoidvoid of ion channels and the multitude of other embedded components, commonly present in the cell membrane. The simplest model of a cell membrane patch is a planar lipid bilayer. The chemical composition of the planar lipid bilayer can be chosen in advance and is therefore well defined. However, to mimic a non-curved fragment of the cell membrane, the planar lipid bilayer should separate two electrolytes. Therefore, the planar lipid bilayer is usually vertically formed across a small aperture in a hydrophobic partition that separates two compartments filled with electrolytes [1]. Visual observation of the planar lipid bilayer in such an experimental system is limited. However, electrodes immersed in the electrolyte permit measurements of electric parameters of the planar lipid bilayer. As a planar lipid bilayer can be electrically considered as a non-perfect capacitor, the capacitance *C* of an ideal capacitor and the resistance *R* of a resistor in parallel both describe planar lipid bilayer’s characteristic [2,3]. Voltage-controlled and current-controlled methods enable observing electrical properties of the planar lipid bilayer and its structural changes that are reflected in the electrical characteristics [4,5,6,7].

The capacitance *C* is the parameter considered as the best tool for probing the stability and proper formation of planar lipid bilayer and for this reason, it is measured for each planar lipid bilayer studied with electrical methods, even when other properties are in the focus of investigation. For comparison between different studies, the measured value of the capacitance is normalized by the area of planar lipid bilayer to determine the specific capacitance *c* [8].

An exposure of planar lipid bilayer to external electrical stimulus and formation of transmembrane voltage alter both electrical parameters of planar lipid bilayer model, *R* and *C*. Molecular dynamics (MD) simulations have shown that small so-called water fingers are formed on both sides of the planar lipid bilayer in the presence of transmembrane voltage and create water or hydrophobic pores [9,10]. The lipids adjacent to the water molecules inside the pores start reorienting their polar headgroups toward water molecules and stabilizing the pores into hydrophilic state, which allows more water and ions to enter the pores. These conductive paths reduce *R* of the planar lipid bilayer. At low transmembrane voltages, when pores are not created yet, a capacitance increase can be observed. An electrostrictive thinning of the planar lipid bilayer has been suggested as the mechanism responsible for this phenomenon [11,12]. The capacitance of the planar lipid bilayer depends on applied voltage *U* according to the equation

(1) $$C\left(U\right)=C_{0}\left[1+\alpha U^{2}\right],$$

where $C_{0}$ is the planar lipid bilayer capacitance at zero transmembrane voltage, and α is the proportionality coefficient. Experiments revealed that its value is around $0.02\;V^{-2}$ [11], while using numerical models it was assessed to be in the range of 0.053–0.082 $V^{-2}$ [13]. However, at higher transmembrane voltages, appearance of water and hydrophilic pores leads to reduction of the planar lipid bilayer capacitance, due to significant difference in dielectric constant values of lipid bilayer and water [14,15]. A long-lasting exposure to strong electric stimulus causes irreversible damage to a planar lipid bilayer. Electrical measurements permit determination of the breakdown voltage value $U_{br}$.

Hydrophobic pores formed in planar lipid bilayer were first hypothesized decades ago in Abidor’s theory of electroporation [16] and as water pores successfully modeled by molecular dynamic simulation [17]. Since such water pores are very small transient structures, they cannot be directly observed. As far as we know, the only study that associated experimental data with the existence of hydrophobic pores in the planar lipid bilayers were reported by Anosov et al. [18]. The experimentally observed increase in the variance of membrane current, that was attributed to small current fluctuations due to membrane capacitance changes, was connected to the number of hydrophobic pores in the membrane during the phase transition. Even experimental information about conducting hydrophilic pores and their characteristics comes only from indirect measurements, like current fluctuations measurements [19], voltage fluctuations measurements [20,21], or observing voltage drops [22]. Recently, Akimov et al. [15,23] introduced an improved theory on the pore formation, which allows modeling continuous trajectories of pore formation, both in the absence and presence of stress conditions. The theory nicely explains the complex behavior of pores during their formation under lateral tension, even in various loading rates regimes that were studied in giant unilamellar vesicles (GUV) aspiration experiments [24]. Namely, at low loading rates, GUV membrane ruptured at small tensions and, according to theory, membrane breakdown is governed by the pore expansion. On the other hand, at high loading rates, GUV membrane rupture occurs at high values of tensions and is theoretically limited by water pore formation, which is lipid type (curvature) dependent. Akimov’s theory predicts that the planar lipid bilayer, exposed to the electric field, behaves similarly as in the presence of lateral tension.

To explore if different membrane rupture mechanisms can be predicted also from experiments in which transmembrane voltage is raised at various rates, we exposed planar lipid bilayers to linearly rising voltage or current signals of different slopes. Planar lipid bilayers were formed using two types of lipid molecules: 1-pamitoyl 2-oleoyl phosphatidylcholine (POPC), lipid molecules with a zwitterionic head group and almost zero spontaneous curvature, negatively charged 1-pamitoyl 2-oleoyl phosphatidylserine (POPS) molecules with negative spontaneous curvature, as well as mixture of both lipid types in a 1:1 ratio. For each planar lipid bilayer composition, *C* was measured using two different methods—discharge pulse [4,25] and voltage to period converter [26,27]. Changing rates of transmembrane voltage rise and measuring $U_{br}$ and time at which planar lipid bilayer rupture occurs $t_{br}$, we evaluated changes in *C* that correspond to water pores, estimated water pore radius at the rupture moment, and calculated the fraction of planar lipid bilayer area that is occupied by water pores.

## 2. Materials and Methods

### 2.1. Chemicals

The lipids 1-pamitoyl 2-oleoyl phosphatidylcholine (POPC) and 1-pamitoyl 2-oleoyl phosphatidylserine (POPS) were purchased in powder form (Avanti Polar-Lipids Inc., Birmingham, AL, USA). A solution of 10 mg/mL of each lipid was prepared in a 9:1 mixture of hexane and ethanol. A mixture of both lipids in a 1:1 ratio was prepared by pipetting the same amount of both lipid solutions in a small plastic tube and mixing by repeated pipetting immediately before the experiments. The mixture of hexadecane and pentane in the ratio of 3:7 was used for torus formation. The electrolyte was prepared from 0.1 M KCl and 0.01 M Hepes in the ratio of 1:1. A few drops of 1 M NaOH were added to obtain a pH of 7.4.

### 2.2. Experimental Setups

**Figure 1:**
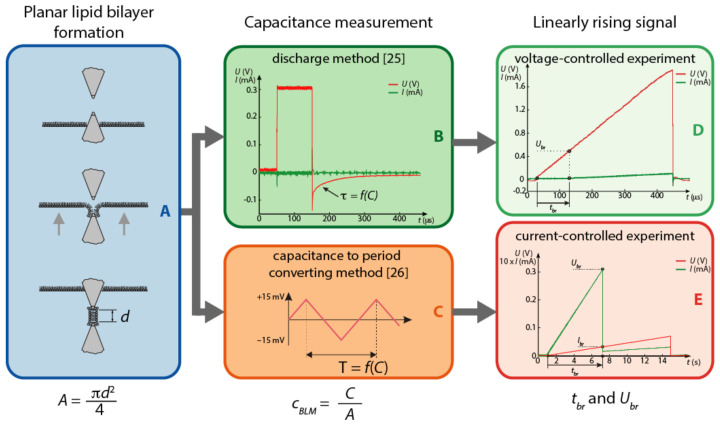
The measurement protocol consisted of formation of the planar lipid bilayer (**A**), capacitance measurement (**B** or **C**), and measurement of the breakdown voltage $U_{br}$ of the planar lipid bilayer (**D** or **E**).

Planar lipid bilayers were formed by the Montal–Muller method [28] across a circular hole 117 m in diameter (d). The hole was in a thin Teflon sheet sandwiched between two reservoirs in the Teflon chamber. The glass window in the Teflon chamber allowed easy viewing of the hole during the formation of the planar lipid bilayer using stereo microscope with led illumination. Both sides of the hole in the Teflon sheet were first treated with 1 L of the lipid solution. After the lipid solvent had completely evaporated, the hole was treated on each side with 1.5 L of the hexadecane and pentane mixture. Both reservoirs were filled with electrolyte just below the hole. A drop of 2 L of the lipid solution was carefully applied to the electrolyte surface in each reservoir and allowed to spread for at least 15 min. To form a planar lipid bilayer, the electrolyte level in both reservoirs was raised to the same height just above the hole (Figure 1A). After a planar lipid bilayer was ruptured, a new one was formed by lowering the electrolyte levels below the hole, and then raising them again. All experiments were performed at room temperature (24 °C).

The voltage-controlled measurement system [29] consisted of a signal generator, two Ag-AgCl electrodes (E-205, IVM, Healdsburg, CA, USA) immersed in the electrolyte, one on each side of the planar lipid bilayer, and amplifiers connected to an oscilloscope for measuring transmembrane current and voltage. The electrodes were used to apply the transmembrane voltage and measure the transmembrane current. The transmembrane voltage was measured using the LeCroy differential amplifier 1822 and custom made ampere meter was used for transmembrane current measurements. Both signals were stored in Matlab format using LeCroy Waverunner-2 354M oscilloscope and analyzed afterwards.

The current-controlled experiments were performed using a potentiostat-galvanostat measurement system controlled by a computer as described in [20]. Four Ag-AgCl electrodes (E-205, IVM, Healdsburg, CA, USA) immersed in the electrolyte were used, two on each side of the planar lipid bilayer. One pair of electrodes was used to apply current and the other was used as a reference electrodes. The transmembrane voltage measurements were stored on a computer in a raw file and later imported into Matlab for further analysis.

### 2.3. Measurement Protocols

The measurement protocol consisted of planar lipid bilayer formation (Figure 1A), capacitance measurement (Figure 1B,C) and measurement of the breakdown voltage of the planar lipid bilayer (Figure 1D,E). *C*, $U_{br}$, and $t_{br}$ were determined for each planar lipid bilayer. The capacitance *C* was normalized to the area of the hole ($A=1.075\times 10^{-8}$ m ${}^{2}$) to calculate the specific capacitance of the planar lipid bilayer $c_{BLM}$. When a voltage-controlled measurement system was used, *C* was determined by the planar lipid bilayer discharge method (Figure 1B) [4]. For current-controlled experiments, *C* was measured by converting the capacitance to the time period (Figure 1C), which was described in detail by Kalinowski et al. [26].

Transmembrane voltage on planar lipid bilayer was buid-up using a linearly rising signal. In the voltage-controlled experiments, rapid transmembrane voltage build-up was ensured by seven different slopes of the linearly rising voltage $k_{u}$: 4.8 kV/s, 5.5 kV/s, 7.8 kV/s, 11.5 16.7 kV/s, 21.6 kV/s, and 48.1 kV/s [25]. At the time $t_{br}$, when a sudden increase in transmembrane current was detected, $U_{br}$ was measured (Figure 1D). In the current-controlled experiments, a slow build-up of the transmembrane voltage was obtained by five different slopes of the linearly rising current $k_{i}$: 0.5 A/s, 1 A/s, 4 A/s, 8 A/s, and 10 A/s. At the time $t_{br}$ when a sudden drop of the voltage was detected, we determined $U_{br}$ (Figure 1E). A linearly rising current signal should produce a quadratic shape of the voltage induced on a planar lipid bilayer, but due to the electrical properties of the experimental system setup, the capacitance and resistance of the chamber flattened the voltage curve.

### 2.4. Experimental Data Analysis

The specific capacitance $c_{BLM}$ was determined for at least 25 planar lipid bilayers of each lipid composition and for each measurement method. Mean value and standard deviation were calculated for each experimental group. Differences in $c_{BLM}$ means between two measurement methods for each lipid composition were determined by t-test. For each lipid composition, at least three measurements of $U_{br}$ were made for each slope of linearly rising voltage and current. To compare $U_{br}$ measured for a given lipid composition at different slopes of the voltage signal, the one-way ANOVA test was used. When comparing the mean breakdown voltages at the same slopes $k_{u}$ or $k_{i}$ between lipid bilayers of one component, the unpaired t-test was used. Since the variances of the mean breakdown voltages at slopes $k_{u}$ = 7.8 kV/s and $k_{u}$ = 11.5 kV/s were statistically different, the comparisons were made using Mann–Whitney’s test. The difference was considered statistically significant at the values of *p* < 0.05.

Using nonlinear regression, a two-parameter strength–duration curve

(2) $$U=\sqrt[{4}]{a+\frac{b}{t}}$$

based on the viscoelastic model of Dimitrov [30] and proposed by Sabotin et al. [31] was fitted to experimentally obtained points $(t_{br}$, $U_{br})$ measured at different slopes of the linearly rising voltage. The parameter $\sqrt[{4}]{a}$ is an asymptote of the strength–duration curve corresponding to minimal breakdown voltage $U_{brmin}$ for planar lipid bilayers of a given lipid composition at fast transmembrane voltage build-up. The parameter *b* determines the inclination of the strength–duration curve. As in the case of strength–duration curve for excitable cell membranes of nerves and muscles, we can define for each lipid composition a kind of a chronaxie time constant $t_{c}=\frac{b}{15\cdot a}$ as the minimum time required for a stimulus of twice $U_{brmin}$ to cause rupture of the planar lipid bilayer.

### 2.5. The Rate of the Planar Lipid Bilayer Capacitance Change at $t_{br}$

Current i(t) which charges planar lipid bilayer can be represented as

(3) $$i\left(t\right)=\frac{u(t)}{R(t)}+\frac{dQ(t)}{dt}=\frac{u(t)}{R(t)}+\frac{d(C(t)u(t))}{dt}=\frac{u(t)}{R(t)}+C\left(t\right)\frac{du(t)}{dt}+u\left(t\right)\frac{C(t)}{dt}.$$

During voltage-controlled experiments, voltage u(t) rises with time linearly in accordance with the slope $k_{u}$. Therefore, Equation (3) changes to

(4) $$i\left(t\right)=\frac{k_{u}}{R(t)}t+C\left(t\right)k_{u}+k_{u}t\frac{dC(t)}{dt}.$$

i(t) at the moment $t_{br}$ is

(5) $$i\left(t_{br}\right)=\frac{k_{u}}{R(t_{br})}t_{br}+C\left(t_{br}\right)k_{u}+k_{u}t_{br}\frac{d(C(t))}{dt}{|}_{t_{br}}.$$

By using experimentally obtained values for $t_{br}$ and $i(t_{br})$, $\frac{d(C(t))}{dt}{|}_{t_{br}}$ can be calculated. We assumed that the changes in *C* due to electrostrictive thinning are small enough to be neglected. Therefore, for each lipid composition, *C* measured before exposure of the planar lipid bilayer to linearly rising signal was used in calculations. As a result that we cannot measure *R* before the membrane breakdown occurs, we calculated it for each lipid composition from current-controlled experiments, performed with the slowest linearly rising current ($k_{i}=0.5$A) at the moment $t_{br}$.

We assumed that the last term in Equation (3) is zero, because *C* has not been changed yet, and the current rises linearly in time according to $i\left(t\right)=k_{i}\cdot t$. Differential equation for transmembrane voltage u(t) was solved using Laplace transform. The solution is

(6) $$u\left(t\right)=k_{i}\cdot R\cdot \left(t-RC+{\left(RC\right)}^{2}\cdot e^{-\frac{t}{RC}}\right),\;t>0.$$

The equation is valid also at the moment $t_{br}$. In the case of slowly linearly rising current, the product RC is at least 10 times smaller than $t_{br}$. Thus, the last summand in the parenthesis in Equation (6) is much smaller than the other two and can be neglected. The Equation (6) can be simplified to

(7) $$C\cdot R^{2}-t_{br}\cdot R+\frac{u(t_{br})}{k_{i}}=0,$$

and solved for *R*.

### 2.6. Calculation of a Fraction of the Planar Lipid Bilayer That Is Occupied By Pores

In the short interval before $t_{br}$ we can assume that non-conductive water pores are already present in the planar lipid bilayer [14,15,32]. Therefore, $C_{br}$ can be represented as

(8) $$C_{br}=\sum_{i=1}^{n}C_{pi}+\sum_{j=1}^{m}C_{lj},$$

where $C_{pi}=\varepsilon_{p}\varepsilon_{0}\frac{A_{pi}}{D}$ is the capacitance of the *i*th water pore and $C_{lj}=\varepsilon_{l}\varepsilon_{0}\frac{A_{lj}}{D}$ capacitance of *j*th patch of the still intact planar lipid bilayer. $\varepsilon_{p}$ is a dielectric constant of water pores ($\varepsilon_{p}$ = 80), $\varepsilon_{l}$ is a dielectric constant of planar lipid bilayer ($\varepsilon_{l}=2$), and $\varepsilon_{0}$ is vacuum permittivity ($\varepsilon_{0}$ = 8.854 $\times 10^{-12}$ Fm ${}^{-1}$). The thickness *D* of the planar lipid bilayer was considered to be37.5 nm and 43.2 nm for POPC and POPS, respectively, obtained from MD simulation as the head to head distance between two electron density profiles [33]. Due to lack of data considering *D* of POPC:POPS mixtures in scientific literature, we determined it according to MD simulation studies which deal with structure, dynamics and hydration dynamics of the mixed lipids [34,35]. Kastel et al. showed presence of domains of POPC and POPS lipids in planar lipid bilayers formed from POPC:POPS (4:1) mixtures [35]. The value 40.0 nm was assumed for the POPC:POPS mixture. Area of the planar lipid bilayer *A* is

(9) $$\sum_{i=1}^{n}A_{pi}+\sum_{j=1}^{m}A_{lj}=A.$$

Therefore, Equation (8) can be rewritten as

(10) $$C_{br}=\frac{\varepsilon_{0}}{D}\left(\varepsilon_{l}A+\sum_{i=1}^{n}A_{pi}\left(\varepsilon_{p}-\varepsilon_{l}\right)\right).$$

Change in planar lipid bilayer capacitance ΔC due to the pore formation is

(11) $$\Delta C=C_{br}-C=-\frac{\varepsilon_{0}}{D}\sum_{i=1}^{n}A_{pi}\cdot \left(\varepsilon_{p}-\varepsilon_{l}\right)),$$

if we assume that capacitance of planar lipid bilayer *C* at the beginning of the experiment is $C=\varepsilon_{l}\varepsilon_{0}\frac{A}{D}$. To obtain the fraction of the planar lipid bilayer that is occupied by pores ($A_{wat}/A$), $\sum_{i=1}^{n}A_{pi}$ is calculated from ΔC and normalized on the whole area of planar lipid bilayer *A*.

We calculated ΔC from $\frac{d(C(t))}{dt}{|}_{t_{br}}$. It was multiplied by the planar lipid bilayer time constant $t_{c}$, which was calculated from experimental data in accordance with Equation (2).

## 3. Results

### 3.1. Experimental Results

The lipid composition $c_{BLM}$, measured by the chosen two methods, was not statistically different (Table 1). Moreover, also the reproducibility of both measuring methods were comparable. Our results, thus, confirm that these two measuring methods are equivalent.

**Figure 2:**
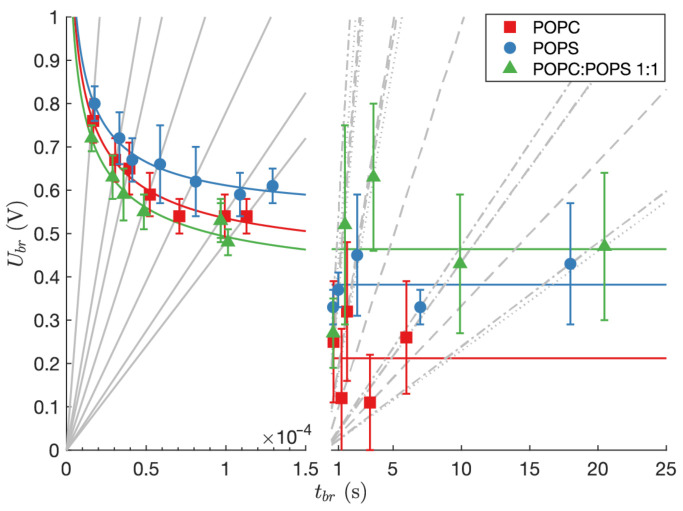
Breakdown voltages of specific lipid compositions measured during voltage-controlled (**left** side) and current-controlled (**right** side) experiments. The gray lines show different slopes ($k_{u}$—**left** side, $k_{i}$—**right** side) of linearly rising signal. Colored curves (**left** side) represent two-parameter strength-duration curves (Equation (2)) fitted to the data obtained by voltage-controlled measurements on POPS, POPC, and POPC:POPS mixture, respectively. Colored lines (**right** side) represent the average values of measured $U_{brI}$ for POPC, POPS, and POPC:POPS mixture, respectively.

Voltage breakdown for each lipid composition was measured by means of linearly rising electrical signal. Fast transmembrane voltage build-up was obtained by voltage-controlled experiments, while for a slow rise of transmembrane voltage current-controlled measurements were used. In Table 2, Table 3 and Table 4, $U_{br}$ values are presented for fast and slow rise of transmembrane voltage, for all three lipid compositions, POPC, POPS, and POPC:POPS mixture, respectively. $U_{br}$ values s, measured using fast rise of transmembrane voltage, follow Equation (2) (Figure 2, left part), while $U_{br}$ values s measured using slow rise of transmembrane voltage exhibit high variability (Figure 2, right part). Therefore, we assumed that $U_{br}$ did not depend on the slope of linearly rising current anymore and simply calculated the mean value of breakdown voltages $U_{brI}$ for each lipid composition, regardless of the linearly rising current slope. The values of $U_{brI}$ were 0.21±0.04 V, 0.38±0.11 V, and 0.46±0.20 V for POPC, POPS, and POPC:POPS mixture, respectively. At these conditions, the $U_{brI}$ values s for planar lipid bilayers of different compositions are not statistically different. At slow rise of transmembrane voltage, the breakdown of planar lipid bilayer occurred in the range of seconds, which is 10${}^{4}$ times slower in comparison with experiments using fast rise of transmembrane voltage (Figure 2). For all lipid compositions $U_{brI}$ was lower than $U_{brmin}$, which was obtained by fitting Equation (2) to the results from experiments with fast rise of transmembrane voltage. The obtained values of $U_{brmin}$ were 0.42±0.01 V, 0.54±0.02 V, and 0.38±0.01 V for POPC, POPS, and POPC:POPS mixture, respectively, while values of $t_{c}$ were 10.4s, 4.5s, 13.1s, presented in the same order.

### 3.2. Modeling Results

**Figure 3:**
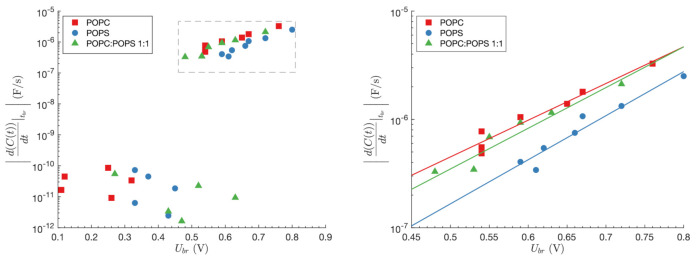
**Left** side of the figure presents the rate of the planar lipid bilayer capacitance change at $t_{br}$ as a function of breakdown voltage $U_{br}$. Results regarding fast rise of transmembrane voltage are enlarged on the **right** side. Slope *m* of the linear relation between $\ln(\frac{dC}{dt})$ and $U_{br}$ is related to pore radius. See text for details.(A) The rate of the planar lipid bilayer capacitance change at $t_{br}$ as a function of breakdown voltage $U_{br}$. Results regarding fast rise of transmembrane voltage are enlarged in (B). Slope *m* of the linear relation between $\ln(\frac{dC}{dt})$ and $U_{br}$ is related to pore radius. See text for details.

The rate of the planar lipid bilayer capacitance change at $t_{br}$ was calculated for all three lipid compositions. The results are presented in Figure 3 (left side). Planar lipid breakdowns that occur at slower transmembrane voltage build-up are accompanied by very small rates of the planar lipid bilayer capacitance change ($10^{-12}\leq \frac{dC}{dt}\leq 10^{-10}$ F/s). They do not follow any specific pattern. While the changes in the planar lipid bilayer capacitance for POPC planar lipid bilayer occur in the 0.11–0.32 V interval and for POPS planar lipid bilayer in 0.33–0.45 V interval, the changes in the planar lipid bilayer capacitance for POPC:POPS mixture are present also at higher voltages (≤0.65 V). The rates of the planar lipid bilayer capacitance change, which accompany fast transmembrane voltage rise, are considerably greater ($10^{-7}\leq \frac{dC}{dt}\leq 10^{-5}$ F/s). As reflected in Figure 3 (right side), the rates of change of the planar lipid bilayer capacitance, in all specific compositions, are exponentially dependent on $U_{br}$: $\ln\left(\frac{dC}{dt}\right)=m\cdot U_{br}+n$. Referring to the kinetic model for membrane failure at fast loading rates, mentioned by Evans et al. [24], the parameter *m* is related to the pore radius *r*, in accordance with the equation $m=\frac{\pi r^{2}}{k_{B}T}$, where $k_{B}$ is Boltzmann constant and *T* is an absolute temperature. Estimated pore radii at the breakdown moments at room temperature (T=298 K), following fast rise of transmembrane voltage were 0.101 nm, 0.110 nm, and 0.106 nm, for planar lipid bilayers composed of POPC, POPS, and POPC:POPS, respectively. Parameter exp(*n*) can be considered as a spontaneous rate of the capacitance change or a spontaneous rate of water pores formation [24]. Its calculated value is 9.1 · 10${}^{-9}$ F/s, 1.5 · 10${}^{-9}$ F/s, and 4.6 · 10${}^{-9}$ F/s for planar lipid bilayers composed of POPC, POPS, and POPC:POPS, respectively.

**Figure 4:**
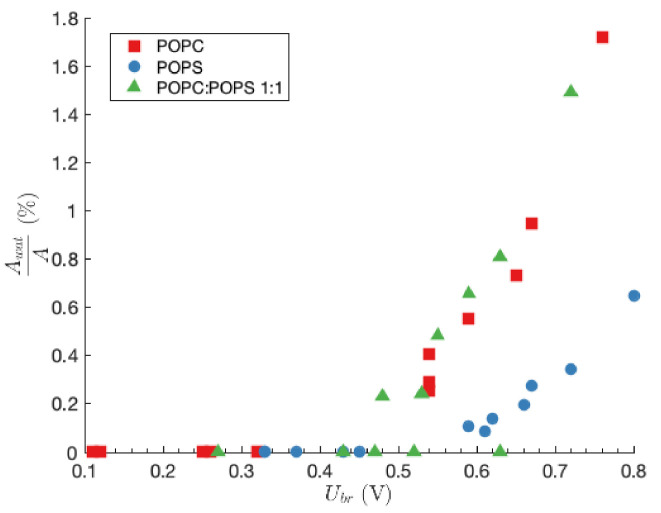
The fraction of the planar lipid bilayer area that is occupied by water pores as a function of $U_{br}$.

In Figure 4, the fraction of the planar lipid bilayer area that is occupied by water pores is presented as a function of $U_{br}$. In the case of slow transmembrane voltage rise, which results in low values of $U_{br}$, the area of the planar lipid bilayer that is occupied by water pores is in the range of $10^{-6}$%.The fraction increases to a couple of percent in case of fast transmembrane voltage rise, which results in higher values of $U_{br}$ (see also data in Table 2, Table 3 and Table 4).

## 4. Discussion

In this study, planar lipid bilayers were exposed to linearly rising voltage and current signals of various slopes to examine $U_{br}$, the change in *C* at $t_{br}$ and the fraction of the planar lipid bilayer occupied by water pores close to the membrane rupture. Three planar lipid bilayer compositions were used; non-charged zwitterionic POPC with mostly zero spontaneous curvature, negatively charged POPS with negative spontaneous curvature, and a mixture of both lipids at 1:1 ratio.

According to the fact that special attention should be given to *C* of each planar lipid bilayer, which is included in experimental studies [8], we measured *C* by two different measuring methods. Using a widely established discharge method and voltage to period conversion method, we proved that both methods give the same results and their reproducibility is comparable.

We found that within the experimental error, the specific capacitance of the POPC planar lipid bilayer was quite similar (Table 1) to that obtained for POPC bilayers reported previously ($c_{BLM}\approx$ 0.6 F/cm ${}^{2}$) [36,37,38], although much lower values ($c_{BLM}$ = 0.28 F/cm ${}^{2}$) have also been reported [39]. In addition, the specific capacitance measured for POPS planar lipid bilayers was slightly lower than values reported in the literature for lipids of similar type ($c_{BLM}$ = 0.624 F/cm ${}^{2}$) [40]. The measured specific capacitance for planar lipid bilayers formed from a POPC:POPS mixture was considerably lower than previously reported values ($c_{BLM}$ = 0.6 F/cm ${}^{2}$) [41]. For all three lipid compositions, we measured lower values of the specific capacitance. The most likely due to the use of a small diameter hole (*d* = 117 m), where the torus is not negligible and therefore the true planar lipid bilayer area is smaller than the geometric area associated with the hole diameter. Micelli et al. [42] studied the capacitance dependence on the hole radius and gave a simple relation for the width of the torus evaluation. If we consider $c_{BLM}$ = 0.6 F/cm ${}^{2}$ as an appropriate value for the specific capacitance of POPC:POPS planar lipid bilayers, the width of the torus is 16.3 m, which is reasonable. We think that the width of the torus can be dependent also on the planar lipid bilayer composition due to lipid molecules packing differences.

From $U_{br}$ measurements presented in Figure 2, it is clear that $U_{br}$ depends on dynamics of transmembrane voltage build-up. Application of slow slopes of linearly rising stimulus ($k_{i}\leq$ 10 A/s) caused high variability of $U_{br}$. The mean values of $U_{br}$ for specific lipid composition, calculated from all measurements obtained by slow slopes of linearly rising stimulus, were not statistically different. It seems that at slow slopes of linearly rising stimulus, the planar lipid bilayer rupture process, which occurs in the range of seconds, is stochastic to a great extent. At constant voltage applied on the planar lipid bilayer, fluctuations of conductance spikes were noticed [19] and associated with defects in planar lipid bilayer that appear and vanish spontaneously. Similarly, we noticed small voltage drops during constant or slowly linearly rising stimulus and reported about them in our previous studies [22,43,44]. Such conductive defects that appear and due to slowly rising stimulus are able also to vanish, locally discharge the planar lipid bilayer and can be the reason for the high variability of $U_{br}$ in slow slopes of linearly rising stimulus conditions. MD simulations support the occurrence of a hydrophilic pore in planar lipid bilayers made from POPC [22] while POPS planar lipid bilayers exhibited only hydrophobic pores [44]. Although the appearance of a pore in POPS planar lipid bilayers was hydrophobic, ions were driven through the pore and discharged the planar lipid bilayer. Full recovery of the planar lipid bilayer in the case of hydrophilic pores is according to MD simulations tens of times longer as in the case of hydrophobic pores, whose lifetime is only a few nanoseconds. This is consistent with experimentally noticed voltage drops in POPS planar lipid bilayers being observed less frequently [44]. Similar observations were reported also on droplet interface planar lipid bilayers, where a population of pores of various sizes were visualized at constant voltage applied [45]. It was also reported that bilayer rupture takes place via a single electropore which grows while other pores shrink and close [45]. The lowest values of $U_{br}$ were obtained for POPC planar lipid bilayers. In some cases of slowly increasing transmembrane voltage, planar lipid bilayers ruptured at transmembrane voltages close to 100 mV, which is in the range of the electric potential difference across a range of biological membranes [46]. Biological membranes, however, are not homogeneous single-component bilayers, but heterogeneous media with a complex organization of lipids and proteins. If we consider planar lipid bilayers of a POPC:POPS (1:1) mixture as a very simple model of a multicomponent bilayer, the experimental results showed that $U_{br}$ is always higher than 200 mV, which can be an approximation of the upper value of the resting potential of biological membranes [47].

Application of fast slopes of linearly rising stimulus (4.8 kV/s $\leq k_{u}\leq$ 48.1 kV/s) resulted in typical “strength-duration” behavior of $U_{br}$ The steeper the slope of the linearly rising stimulus, the higher the transmembrane voltage that is needed for planar lipid bilayer rupture. We fitted the strength–duration curve proposed by Dimitrov [30] to ($t_{br}$, $U_{br}$) points measured for all three lipid compositions. The chosen strength–duration curve considers the planar lipid bilayer as a thin viscoelastic film with fluctuating surfaces and was originally used to predict the critical voltage and critical time needed to breakdown the planar lipid bilayer at applied constant voltage. Planar lipid bilayers composed of POPS ruptured at higher voltages than planar lipid bilayers composed of POPC or the POPC:POPS mixture. According to the study on POPS/POPC (1:20) vesicles [48] and according to the study on solid-supported membranes of POPC:POPS (4:1) [35], irregularly shaped domains are formed in bilayers of POPC-POPS lipid mixtures. The formation of anionic lipid domains is mediated by the presence of cations in the environment; experiments and MD have shown that Ca ${}^{2+}$ ions force the formation of larger PS domains [49] and it is also known that monovalent ions are able to induce domain formation [50]. However, charged lipids themselves also form segregated zones in membranes [51]. Moreover, the phase transition temperatures of PS lipids are always higher than those of its PC counterparts with the same acyl chains (e.g., that of POPC is −2 °C and that of POPS is +14 °C [52]), so incomplete lateral mixing of POPC and POPS is expected.

Measurements based on 2H nuclear magnetic resonance [48,53] have shown that the lipid hydrocarbon chains in pure PC and PS membranes have different order parameters at room temperatures. Namely, although both were in a lamellar liquid crystalline phase, PS was more ordered than PC. This difference is also reflected in differences in the lipid–lipid interaction at the lipid–water interface; namely, in the presence of an electric field, the incorporation of water into the hydrophobic region of the planar lipid bilayer is significantly enhanced in the disordered domains and almost unchanged in the ordered domains [54]. From molecular dynamics simulation studies, two regions in bilayers consisting of different domains are suggested for pore formation in the presence of an electric field: lipid regions with greater disorder [54,55] and thinned regions at domain boundaries [56]. According to Reigada et al. [54], pores are formed in lipid regions with greater disorder, and the smaller the disordered domains, the faster they are electroporated.. Our results are in line with these observations. Indeed, $U_{brmin}$ were 0.42±0.01 V and 0.54±0.02 V for POPC and POPS planar lipid bilayers, respectively, implying that pores form more easily in more disordered POPC planar lipid bilayers. Moreover, for POPC planar lipid bilayers, the estimated spontaneous rate of water pore formation is six times higher than in the case of POPS planar lipid bilayers. $U_{brmin}$ obtained for POPC:POPS mixture was even lower, 0.38±0.01 V, which is consitent with the fact that smaller disordered domains are more susceptible for pore formation. On the other hand, the formation of pores could also be expected at the domain boundaries, where near the phase transition of single-component DPPC planar lipid bilayer, pore formation induced by an external electric field was obtained in thinned regions at the liquid and gel-domain interfaces [56]. Although our experiments were performed far from the transition temperature (at least 10 °C), thinner regions with reduced stability [57] can also be attributed to boundaries between disordered POPC and more ordered POPS lipid domains.

The rate of capacitance change of the planar lipid bilayer can be related to the rate of water pores formation [14,16,18]. Ruptures of planar lipid bilayer that occur as a consequence of a slow build-up of transmembrane voltage are accompanied by very small rates of the capacitance change, which do not exhibit any specific pattern. However, the rates of capacitance change which appear at fast build-up of transmembrane voltage are exponentially dependent on $U_{br}$. Similarly, Evans et al. [24] reported that the size and frequency of initial defect formation of fluid membrane vesicles depend on the loading rate of micro-pipette suction. Estimated radii of pores that govern ruptures of a planar lipid bilayer are 0.101 nm, 0.110 nm, and 0.106 nm for planar lipid bilayers composed of POPC, POPS, and POPC:POPS, respectively. The values are in the range of radii for hydrophobic pores predicted by Akimov’s model [15]. The model also predicts a slightly larger radius of hydrophobic pores for planar lipid bilayers composed of lipids with negative spontaneous curvature while compared with planar lipid bilayers composed of lipids with zero spontaneous curvature, which is in agreement with our experiments and modeling.

Estimation of the area fraction that is occupied with water pores at the moment of planar lipid bilayer rupture reveals that the number of water pores is considerably lower in the case of slow build-up of transmembrane voltage. It is probably due to the fact that during slow rise of the transmembrane voltage, conducting pores are already present in planar lipid bilayer [19,45], which also changes the electrical resistance of planar lipid bilayer. It should be noted that this change was not taken into account in our calculations. Aqueous fractional area in the planar lipid bilayer due to an exponential pulse, bipolar square pulse, or a square pulse was theoretically evaluated by Freeman et al. in 1994 [14]. According to their calculations, only 0.1% of the membrane is occupied by pores, which is in good agreement with our results obtained for a fast build-up of transmembrane voltage. Moreover, recently, Anosov et al. [18] presented a computational model which predicts the number of hydrophobic pores in planar lipid bilayer using the variance of the membrane current. Their calculations showed that the area of hydrophobic pores at the phase transition (absence of a stimulus) does not exceed 4.5% of the planar lipid bilayer area, which is also in agreement with our observations.

The area fraction that is occupied with water pores is similar in the case of planar lipid bilayers formed of POPC and POPC:POPS mixture, while POPS planar lipid bilayers exhibited lower values. Lipid molecules in POPS planar lipid bilayers are more ordered than in POPC planar lipid bilayers [58] and according to MD simulations in POPS planar lipid bilayers mostly hydrophobic pores are formed where lifetime is only a few nanoseconds [44], allowing quick planar lipid bilayer discharge. As a result that pores are harder to form in ordered planar lipid bilayer, also our results show a six times lower spontaneous rate of water pore formation in comparison with POPC lipids. Therefore, it is possible that the area fraction that is occupied with water pores at the moment of planar lipid bilayer rupture in POPS planar lipid bilayers is indeed noticeably smaller in comparison with POPC and POPC:POPS planar lipid bilayers. The area fraction occupied with water pores at the moment of the planar lipid bilayers rupture is similar in the case of POPC and POPC:POPS planar lipid bilayers. Although it seems that in the planar lipid bilayers made of POPC:POPS mixture, pores are predominantly formed in more disordered POPC domains, additional experimental and theoretical studies are needed to confirm this guess.

Estimation of the radius of a water pore that governs planar lipid bilayer rupture and the fraction of planar lipid bilayer area that is occupied by water pores at the moment of rupture is based on prediction that small water pores are present in the planar lipid bilayer. Therefore, the difference in the dielectric permittivity of planar lipid bilayer (ε = 2) and water (ε = 80) is the only reason for the change in capacitance. This assumption is a considerable simplification of all phenomena that are involved in water pore creation and existence. Close to the charged surface, for example, strong orientational water dipole ordering may result in a local decrease of permittivity [59,60]. The relative permittivity of the electrolyte solution decreases with the increasing magnitude of the electric field strength. In narrow tubes, like in water pores, the water dipoles are partially oriented also close to the axis of the tube, which decreases the relative water permittivity [61]. To obtain more reliable results, at least orientational ordering of water molecules in pores and close to the membrane should be taken into account.

## 5. Conclusions

Theories of pore formation and molecular dynamics simulations suggest recurrent hydrophobic pores or water pores whose occurrence is strongly dependent on the electric field. Experimental information about them is very rare. In our study, planar lipid bilayers of POPC, POPS, and a POPC:POPS (1:1) mixture were exposed to different slopes of linearly increasing voltage or current signal. Such experiments allowed to determine the breakdown voltage of planar lipid bilayers, and taking into account that the membrane can be represented by an RC circuit, the fraction of the planar lipid bilayer occupied by water pores could be calculated. Our results confirm the results of pore formation theories and molecular dynamics models. The fraction of area occupied by water pores at the moment of planar lipid bilayer rupture is considerably lower when the transmembrane voltage is built up slower than in the case of a fast build-up of transmembrane voltage. In more disordered planar lipid bilayers, water pores are easily formed, and the radii of the water pores are smaller in this case than in more ordered planar lipid bilayers. In the case of lipid mixtures, water pores are formed even more easily, probably due to smaller disordered domains in the planar lipid bilayer or/and weaker regions at the domain boundaries.

## Figures and Tables

**Table 1:** Specific capacitance of planar lipid bilayers ($c_{BLM}$) measured by discharge and capacitance to a period converting method. The values are given as mean ± standard deviation (number of measurements).

Lipid Mixtures	Discharge Method [25] $c_{\mathrm{BLM}}$ [F/cm ${}^{2}$]	Capacitance to Period Converting Method [26] $c_{\mathrm{BLM}}$ [F/cm ${}^{2}$]
POPC	0.51 ± 0.17 (80)	0.51 ± 0.16 (58) *
POPS	0.41 ± 0.14 (76)	0.41 ± 0.13 (34) *
POPC:POPS 1:1	0.31 ± 0.07 (60)	0.34 ± 0.17 (25) *

* *p* > 0.05.

**Table 2:** Results for planar lipid bilayers composed of POPC. In the upper part of the table, the results of voltage-controlled measurements with seven different slopes $k_{u}$ are gathered while the results of current-controlled measurements with five different slopes $k_{i}$ are presented in the lower part of the table. For each slope of linearly rising signal, the planar lipid breakdown voltage $U_{br}$ and lifetime $t_{br}$ are presented. Values are given as means ± standard deviations. $\frac{A_{wat}}{A}$ is a calculated fraction of the planar lipid bilayer that is occupied by pores. *N* is the number of experiments in each experimental group.

POPC					
	$k_{u}$ **(kV/s)**	N	$U_{\mathrm{br}}$ **(V)**	$t_{\mathrm{br}}$ **(s)**	$\frac{A_{\mathrm{wat}}}{A}$ **(%)**
voltage-cont.	48.1	16	0.76 ± 0.05	16.76 ± 1.14	1.72
	21.6	17	0.67 ± 0.05	30.50 ± 2.60	0.95
	16.7	12	0.65 ± 0.06	39.38 ± 3.87	0.73
	11.5	12	0.59 ± 0.05	52.25 ± 4.65	0.55
	7.8	16	0.54 ± 0.04	70.74 ± 5.07	0.41
	5.5	16	0.54 ± 0.05	99.24 ± 9.93	0.29
	4.8	17	0.54 ± 0.04	112.92 ± 8.32	0.27
	$k_{i}$ **(A/s)**	N	$U_{\mathrm{br}}$ **(V)**	$t_{\mathrm{br}}$ **(s)**	$\frac{A_{\mathrm{wat}}}{A}$ **(%)**
current-cont.	10	8	0.25 ± 0.14	0.64 ± 0.55	45.20 $\times 10^{-6}$
	8	9	0.12 ± 0.16	1.22 ± 1.16	23.63 $\times 10^{-6}$
	4	7	0.32 ± 0.16	1.63 ± 1.35	17.76 $\times 10^{-6}$
	1	11	0.11 ± 0.11	3.30 ± 0.98	8.74 $\times 10^{-6}$
	0.5	16	0.26 ± 0.13	5.97 ± 2.09	4.83 $\times 10^{-6}$

**Table 3:** Results for planar lipid bilayers composed of POPS. In the upper part of the table, the results of voltage-controlled measurements with seven different slopes $k_{u}$ are gathered while the results of current-controlled measurements with five different slopes $k_{i}$ are presented in the lower part of the table. For each slope of linearly rising signal the planar lipid breakdown voltage $U_{br}$ and lifetime $t_{br}$ are presented. Values are given as means ± standard deviations. $\frac{A_{wat}}{A}$ is a calculated fraction of the planar lipid bilayer that is occupied by pores. *N* is the number of experiments in each experimental group.

POPS					
	$k_{u}$ **(kV/s)**	N	$U_{\mathrm{br}}$ **(V)**	$t_{\mathrm{br}}$ **(s)**	$\frac{A_{\mathrm{wat}}}{A}$ **(%)**
voltage-cont.	48.1	18	0.80 ± 0.04	17.59 ± 0.86	0.65
	21.6	14	0.72 ± 0.06	33.15 ± 2.76	0.35
	16.7	13	0.67 ± 0.05	41.24 ± 3.34	0.28
	11.5	12	0.66 ± 0.09	58.66 ± 7.71	0.20
	7.8	15	0.62 ± 0.08	81.05 ± 10.92	0.14
	5.5	13	0.59 ± 0.05	108.78 ± 8.72	0.11
	4.8	15	0.61 ± 0.04	129.24 ± 7.86	0.09
	$k_{i}$ **(A/s)**	N	$U_{\mathrm{br}}$ **(V)**	$t_{\mathrm{br}}$ **(s)**	$\frac{A_{\mathrm{wat}}}{A}$ **(%)**
current-cont.	10	5	0.33 ± 0.04	0.61 ± 0.26	18.91 $\times 10^{-6}$
	8	5	0.37 ± 0.04	0.98 ± 0.08	11.72 $\times 10^{-6}$
	4	6	0.45 ± 0.14	2.37 ± 0.71	4.84 $\times 10^{-6}$
	1	5	0.33 ± 0.04	6.99 ± 0.91	1.64 $\times 10^{-6}$
	0.5	6	0.43 ± 0.14	17.98 ± 5.85	0.64 $\times 10^{-6}$

**Table 4:** Results for planar lipid bilayers composed of POPC:POPS. In the upper part of the table, the results of voltage-controlled measurements with seven different slopes $k_{u}$ are gathered while the results of current-controlled measurements with five different slopes $k_{i}$ are presented in the lower part of the table. For each slope of linearly rising signal, the planar lipid breakdown voltage $U_{br}$ and lifetime $t_{br}$ are presented. Values are given as means ± standard deviations. $\frac{A_{wat}}{A}$ is a calculated fraction of the planar lipid bilayer that is occupied by pores. *N* is the number of experiments in each experimental group.

POPC:POPS 1:1					
	$k_{u}$ **(kV/s)**	N	$U_{\mathrm{br}}$ **(V)**	$t_{\mathrm{br}}$ **(s)**	$\frac{A_{\mathrm{wat}}}{A}$ **(%)**
voltage-cont.	48.1	7	0.72 ± 0.03	15.73 ± 0.73	1.49
	21.6	7	0.63 ± 0.05	28.90 ± 2.30	0.81
	16.7	6	0.59 ± 0.06	35.73 ± 3.70	0.66
	11.5	7	0.55 ± 0.04	48.49 ± 3.55	0.48
	7.8	11	0.53 ± 0.04	96.66 ± 4.60	0.24
	5.5	9	0.53 ± 0.05	96.68 ± 9.12	0.24
	4.8	13	0.48 ± 0.03	101.24 ± 7.39	0.23
	$k_{i}$ **(A/s)**	N	$U_{\mathrm{br}}$ **(V)**	$t_{\mathrm{br}}$ **(s)**	$\frac{A_{\mathrm{wat}}}{A}$ **(%)**
current-cont.	10	3	0.27 ± 0.08	0.61 ± 0.19	15.92 $\times 10^{-6}$
	8	5	0.52 ± 0.23	1.47 ± 0.51	38.47 $\times 10^{-6}$
	4	5	0.63 ± 0.17	3.56 ± 0.94	6.59 $\times 10^{-6}$
	1	6	0.43 ± 0.16	9.90 ± 3.70	2.37 $\times 10^{-6}$
	0.5	5	0.47 ± 0.17	20.47 ± 8.92	1.15 $\times 10^{-6}$

## Data Availability

All data presented are available in this article.

## Abbreviations

The following abbreviations are used in this manuscript:

POPC	1-pamitoyl 2-oleoyl phosphatidylcholine
POPS	1-pamitoyl 2-oleoyl phosphatidylserine
MD	Molecular dynamics simulation
GUV	Giant unilamellar vesicles
